# Low Plasma Levels of Hyaluronic Acid Might Rule Out Sinusoidal Obstruction Syndrome after Hematopoietic Stem Cell Transplantation

**DOI:** 10.1155/2023/7589017

**Published:** 2023-04-17

**Authors:** Carmen De Ramón Ortiz, Raul Justo Sanz, Yan Beauverd, Karem Humala, Ana López de la Guia, Raquel De Paz, Mercedes Gasior, Pilar Gómez Prieto, Marta Fabra Urdiola, Miguel Canales Albendea, Nora Butta, Victor Jiménez Yuste

**Affiliations:** ^1^Hematology, University Hospital of Geneva, Switzerland; ^2^Hematology, Hospital La Paz Institute for Health Research, Madrid, Spain; ^3^Hematology, La Paz University Hospital, Madrid, Spain

## Abstract

**Background:**

Sinusoidal obstructive syndrome (SOS) is a potentially fatal complication secondary to hematopoietic stem cell transplant (HSCT) conditioning. Endothelial damage plasma biomarkers such as plasminogen activator inhibitor-1 (PAI-1), hyaluronic acid (HA), and vascular adhesion molecule-1 (VCAM1) represent potential diagnostic tools for SOS.

**Methods:**

We prospectively collected serial citrated blood samples (baseline, day 0, day 7, and day 14) in all adult patients undergoing HSCT at La Paz Hospital, Madrid. Samples were later analyzed by ELISA (enzyme-linked immunosorbent assay) for HA, VCAM1, and PAI-1 concentrations.

**Results:**

During sixteen months, we prospectively recruited 47 patients. Seven patients (14%) were diagnosed with SOS according to the EBMT criteria for SOS/VOD diagnosis and received treatment with defibrotide. Our study showed a statistically significant elevation of HA on day 7 in SOS patients, preceding clinical SOS diagnosis, with a sensitivity of 100%. Furthermore, we observed a significant increase of HA and VCAM1 levels on day 14. Regarding risk factors, we observed a statistically significant association between SOS diagnosis and the fact that patients received 3 or more previous lines of treatment before HSCT.

**Conclusions:**

The early significant increase in HA levels observed opens the door to a noninvasive peripheral blood test which could have the potential to improve diagnosis and facilitate prophylactic and therapeutic management of SOS before clinical/histological damage is established.

## 1. Background

Sinusoidal obstructive syndrome (SOS) or venooclusive disease (VOD) is a complication of hematopoietic stem cell transplant (HSCT) which derives from the endothelial damage provoked by conditioning regimens. It is associated with hepatomegaly, ascites, jaundice, and thrombocytopenia and can progress to multiorgan failure (MOD) and death [1, 2].

Classical SOS typically occurs between the first and third weeks following HSCT, but it may also occur later. When promptly diagnosed and treated, SOS is reversible in most patients, but its mortality rate could exceed 80% in the presence of MOD [3]. A decrease in the incidence of SOS has been reported in the last few years [4]. However, recent evidence suggests that SOS might be underdiagnosed, especially when occurring in a late onset setting [5, 6].

To date, SOS diagnosis is made according to clinical findings [7]. In the adult setting, the most recent criteria are the EBMT criteria for SOS/VOD diagnosis in adults [8] which require the presence of hyperbilirubinemia (total bilirubin ≥ 2 mg/dl) and at least two of the following: painful hepatomegaly and >5% weight gain or ascites within 3 weeks of HSCT [9]. Abdominal ultrasound (US) showing a reversal of the sinusoidal flow among other abnormalities is commonly used to support SOS diagnosis. Nevertheless, it requires an expert sonographer and its role is still controversial because of nonuniformity of results [10, 11]. Recently, novel simplified US scoring systems have been described (HokUS-10 and HokUS-6) [12–15] which show an excellent diagnostic value for SOS but still need to be validated in larger and prospective trials. Transjugular liver biopsy for histological evidence of SOS remains the gold standard but is invasive and difficult to perform and is therefore not mandatory for diagnosis [8, 16, 17].

However, all these findings are late events in the pathology of the disease [18] and emerge after histological damage is established [19]. Prophylaxis and treatment with defibrotide (Jazz Pharmaceutical, Palo Alto, CA) have shown the most promising results in several clinical trial [20–23]. Nevertheless, treatment with defibrotide can carry significant risks, such as severe hemorrhage, especially in the context of severe thrombocytopenia present in this patient population [24–26]. Therefore, there is a clear need for a noninvasive and accurate method for early SOS diagnosis [2, 18].

There is still no validated blood test to confirm SOS diagnosis. Several potential biomarkers have been suggested, such as hyaluronic acid (HA), vascular adhesion molecule-1 (VCAM1), and plasminogen activator inhibitor-1 (PAI-1) [27]. However, to date, none of these biomarkers have shown a significant increase before the clinical SOS diagnosis was established. Systemic levels of these molecules are regarded as possible markers of hepatic sinusoidal endothelial cell damage [27–29].

In addition, a biomarker panel called EASIX (“endothelial activation and stress index”) [29], containing creatinine, lactate dehydrogenase, and low platelets, has recently been described a potential surrogate for predicting endothelial injury before conditioning and day 0 [30]. Another clinical risk score published by the Center for International Blood and Marrow Transplant Research (CIBMTR) [31] can potentially predict the development of SOS.

Following HSCT, injury to the endothelium induces inflammation which triggers the release of multiple coagulation factors, adhesion molecules (such as VCAM1) and also fibrinolysis inhibitors (such as PAI-1) [9]. Moreover, HA is an extracellular polysaccharide which is elevated in the case of histologic injury in patients with chronic liver disease (cirrhosis and acute cellular rejection in liver transplant patients) [4, 32–34] and also in SOS secondary to oxaliplatin-based chemotherapy [29].

Incidence of SOS is highly associated with the presence of several risk factors related either to the type of transplant and conditioning regimen or to the patient's characteristics such as age or prior therapy [35].

The aim of this prospective observational study is to evaluate plasma HA, VCAM1, and PAI-1 as diagnostic biomarkers in patients with SOS following HSCT and to correlate serial measurements of these biomarkers with the evolution of SOS.

## 2. Methods

All adult patients (≥18 years old) who underwent an allogeneic or autologous transplant at La Paz University Hospital in Madrid, Spain, were prospectively included during sixteen months in this unicentric observational study. At our center, approximately 40 patients per year undergo allogeneic or autologous transplant. Given the low reported incidence of SOS that generally ranges from 5 to 20% [2, 36], this study is designed as an exploratory analysis and its results should be later confirmed in a multicentric study with a larger patient population.

According to our local guidelines for SOS prophylaxis, all patients received prophylactic low molecular weight heparin throughout the entire period of hospitalization. Patients were diagnosed with SOS when they fulfilled the EBMT criteria within 3 weeks of HSCT. At diagnosis, patients were started on intravenous defibrotide (Jazz Pharmaceutical) 25 mg/kg/day.

Citrated peripheral blood samples were collected from all patients at four timepoints: the day before the start of the conditioning regimen (baseline measurement), the day of stem cell infusion (day 0), and day 7 and day 14 after infusion. From patients who developed SOS (SOS group), additional samples were obtained on day 21 and once per week until the resolution of SOS or discontinuation of defibrotide. Patients who underwent HSCT without developing SOS were the control group (non-SOS group).

Platelet-free plasma was obtained by double centrifugation at 23°C (15 minutes at 1,500 g and 2 minutes at 13,000 g) and stored at -80°C in aliquots until analysis with ELISA kits of HA (Cusabio, Texas, USA), VCAM1 (Sigma, Madrid, Spain), and PAI-1 eBioscience, Vienna, Austria) concentrations that were performed within the three months after its obtention. Routine laboratory analysis data was also registered. Total bilirubin was tested in serum samples using Siemens reagents in Atellica equipment within two hours after blood draw. HA's normal range is 5.12-92 ng/ml, sensitivity 0.027-0.200 ng/ml, intra-assay precision coefficient of variation % (CV%) 7.2% and 3.6% for low and high concentrations, respectively, and interassay precision CV% 4.8%. VCAM1's normal range is 349-991 ng/ml, sensitivity 300 pg/ml, intra-assay precision CV% < 10%, and interssay precision CV% < 12%. PAI-1's normal range is 0.9-99.0 ng/ml, sensitivity 29.0 pg/ml, intra-assay precision CV% 4.7%, and interassay precision CV% 5.0%. Total bilirubin's normal range is 0.30-1.20 mg/dl, sensitivity <0.1 mg/dl, intra-assay precision CV% 2.1%, and interassay precision CV% 6.3%.

The primary endpoint was the comparison of the plasma level of HA, VCAM1, PAI-1, and total bilirubin between the SOS group and non-SOS group. Secondary endpoints were as follows: (1) the evolution of biomarkers between the SOS group and non-SOS group and (2) the comparison of risk factors between the SOS group and non-SOS group.

Statistical analyses were performed with SPSS software. Receiver operating characteristics (ROC) curve from logistic regression models with area under the curve (AUC) was used to present the correlation of clinical outcomes and biomarkers. Fisher's exact test was used to describe the association between SOS development and known risk factors. A *p* value of ≤0.05 is considered statistically significant.

Study protocol was approved by the Ethics Committee of La Paz University Hospital (PI-1743), and informed consent was obtained from all participating patients. The study was conducted in accordance with the Declaration of Helsinki.

## 3. Results

We prospectively included 47 patients undergoing allogeneic or autologous HSCT at our center. Seven patients (14%) were diagnosed with SOS between days 10 and 15 (median 12 days) after HSCT according to the EBMT criteria and immediately started on intravenous defibrotide. All 7 patients presented with hyperbilirubinemia (total bilirubin ≥ 2 mg/dl) at SOS diagnosis. Abdominal US were performed, showing SOS signs in all 7 patients. No late-onset SOS was objectivated. Transjugular liver biopsy was not performed in SOS patients due to severe thrombocytopenia present in all patients at the time of diagnosis.  Table 1 summarizes patient's characteristics of the whole study population.

Biomarker data were available, as planned, at baseline, on days 0, 7, 14, and 21 in all 7 SOS patients. Additional samples were obtained on day 28 in 6 of the patients, who were still on defibrotide by that time. Regarding the non-SOS group, for logistic reasons, biomarker data for day 7 were only available in the first 16 patients included in the study (8 patients undergoing allogeneic and 8 autologous HSCT). For the other planned timepoints (baseline, day 0, and day 14), biomarker results were available for 35 of the 40 of non-SOS patients.

**Figure 1:**
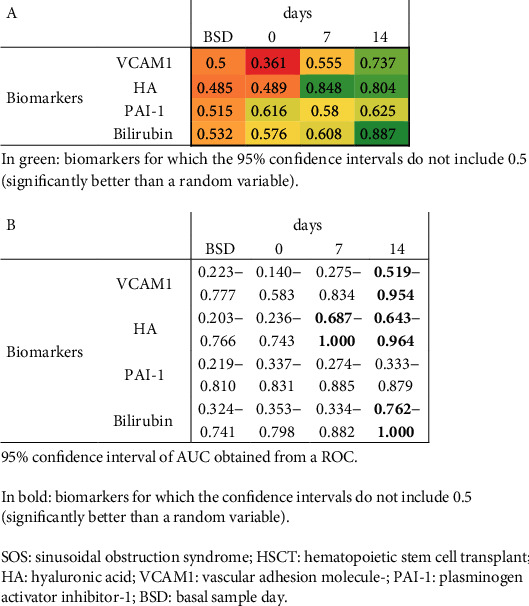
Association of each biomarker with the development of SOS at different times of HSCT. AUC (area under the curve) obtained from a ROC (receiver operating characteristic).

**Figure 2:**
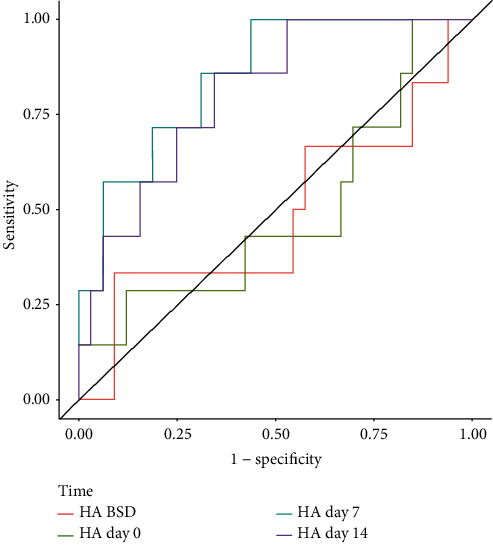
ROC curves of HA predicting SOS for different times of HSCT. SOS: sinusoidal obstruction syndrome; HSCT: hematopoietic stem cell transplant; HA: hyaluronic acid; ROC: receiver operating characteristic.

In the SOS group, HA significantly increased on day 7 (AUC = 0.848; 95% CI: 0.687-1.000) (Figure 1), which is remarkable because it took place before the clinical SOS diagnosis. On day 14, HA was still significantly elevated (AUC = 0.804; 95% CI: 0.643-0.964).  Figure 2 represents the ROC curves for HA at different times. With a cut-off value of 172 ng/ml, HA sensitivity for SOS diagnosis is 100% (95% CI: 59.04-100.00), while specificity is 56.25% (95% CI: 29.88-80.25) (Table 2). Positive predictive value is 50%, and negative predictive value is 100%. The cut-off value of 172 ng/ml was chosen as this was the highest value still yielding 100% sensitivity within the sample. The other biomarkers analyzed show no statistically significant increase at day 7 (Figure 1).

When analyzing patients undergoing allogeneic and autologous HSCT separately, we observed that HA is significantly increased on day 7 in allogeneic HSCT patients (AUC = 0.875; 95% CI: 0.663-1) with a 100% sensitivity for SOS diagnosis (95% CI: 54 to 100) and a 75% specificity (95% CI: 35-97) (Supplementary Figure 1 and Supplementary Table 1). Given the fact that only one patient developed SOS in the autologous HSCT group, results are not statistically significant for this group (100% sensitivity (95% CI: 2-100) and 37% specificity (95% CI: 9-76) for SOS diagnosis for HA at day 7). We observed similar HA baseline level and kinetics in both groups (Supplementary Figure 2).

VCAM1 levels (AUC = 0.737; 95% CI: 0.519-0.954) significantly increased on day 14 in the SOS group, along with an increase in total bilirubin (AUC = 0.887; 95% CI: 0.762-1.000). Regarding PAI-1 kinetics in patients who developed SOS, we observed a moderate increase on day 14 which was not statistically significant (Figure 1).

**Figure 3:**
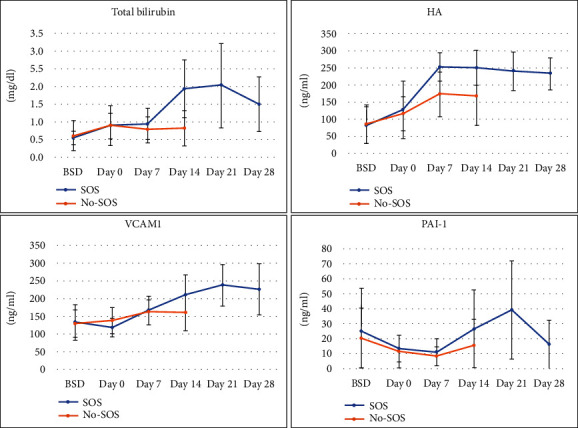
Evolution of the mean (standard deviations) of each biomarker in patients with and without SOS during HSCT. SOS: sinusoidal obstruction syndrome; HSCT: hematopoietic stem cell transplant; HA: hyaluronic acid; VCAM1: vascular adhesion molecule-; PAI-1: plasminogen activator inhibitor-1; BSD: basal sample day.

The outcome of all 7 SOS patients was favorable after treatment with defibrotide. Total bilirubin levels decreased after a median of 7 days of treatment. No significant bleeding was observed during defibrotide treatment despite the severe thrombocytopenia present in all patients. No thrombotic events occurred in either group during our study.  Figure 3 shows the evolution of the biomarkers during the study. In the SOS group, PAI-1 decreased during defibrotide treatment (day 14 to day 28) and tended to return to baseline with SOS resolution. The same occurred to total bilirubin levels, which decreased during treatment and normalized (<2 mg/dl) in all patients by day 28. On the other hand, HA and VCAM1 levels remained stable despite SOS remission.

Finally, we observed an association between SOS diagnosis and known SOS risk factors. Patients who received 3 or more previous lines of treatment before HSCT have a statistically significant risk of developing SOS (71.4% SOS vs. 27.5% non-SOS; *p* = 0.036) (Table 3). Additionally, 66% of the patients who developed SOS had received a haploidentical HSCT, against only 33% of patients at the non-SOS group (*p* = 0.18). Other known SOS risk factors were also present in the SOS group: higher median age (59 vs. 51 years), previous autologous HSCT (28.6% vs. 7.5%, *p* = 0.15), and having received a busulfan-based conditioning (71.4% vs. 55%, *p* = 0.68), but they did not reach statistical significance.

## 4. Discussion

New tools are required to establish early and accurate SOS diagnosis after HSCT [37]. In recent years, there has been significant progress; however, no biomarker is yet available for clinical use in the HSCT setting [16, 38].

In the present study, we have prospectively measured several plasma biomarkers as potential markers for hepatic sinusoidal endothelial cell injury [27]. Consistent with recent studies [32, 33], we have shown an association between the increase of these biomarkers (HA, VCAM1, and PAI-1) and SOS development.

Regarding HA, two previous studies had described a tendency towards an increase in HA levels in patients who develop SOS, but no significant results at an early stage of transplant being described so far [27, 28]. HA is normally cleared by hepatic sinusoidal endothelial cells. When these cells are injured in SOS, there is decreased clearance of HA which is the potential explanation for this increase [27, 29]. Herein, we have observed for the first time a statistically significant elevation of HA on day 7 and day 14. Interestingly, the rise in plasma HA levels on day 7 preceded the rise of total bilirubin and clinical SOS diagnosis in all 7 patients.

When using 172 ng/ml as a cut-off, the sensitivity and specificity of plasma HA on day 7 for the diagnosis of SOS are 100% (95% CI: 59.04-100.00) and 56% (95% CI: 29.88-80.25), respectively, and the positive and negative predictive values were 50% and 100%, respectively. Our data suggests that absence of HA elevation on day 7 could rule out future development of SOS. It could be hypothesized that those patients might not require defibrotide prophylaxis, while we could discuss defibrotide prophylaxis in the rest of cases in order to avoid progression to overt SOS [29]. This early significant increase in HA plasma levels could represent a simple and noninvasive method to accelerate SOS diagnosis before a histological damage has occurred and before SOS symptoms are overt [24]. Nevertheless, given the small number of patients in our study, large multicentric trials are needed to validate these results and to define a cut-off for plasma HA levels that could be used in regular practice [27, 33].

Furthermore, when looking into allogeneic and autologous HSCT separately, HA elevation on day 7 is statistically significant for allogeneic HSCT patients, with a 100% sensitivity (95% CI: 54-100). Given the small number of patients undergoing autologous HSCT included in the study, no conclusions can be specifically drawn from this subgroup of patients. We observed a similar behavior of HA kinetics and baseline level in both allogeneic and autologous HSCT. However, our study was not designed to compare both populations of patients and larger studies are needed to confirm whether this biomarker could be useful in both settings.

VCAM1 is a sialoglycoprotein present on the cell surface of cytokine-activated endothelium that participates in the adhesion of leukocytes to endothelium [39]. The role of VCAM1 in SOS development has been previously reported. Consistent with a previous study, we observed a significant increase of VCAM1 levels on day 14 [27, 40] when the EBMT criteria were already met.

Levels of PAI-1, a fibrinolysis suppressor, increase in the presence of thrombosis and in the presence of other diseases such as septic shock [27] and probably SOS [41]. In our study, we did not observe significant differences in PAI-1 levels between patients with or without SOS. A moderate increase in PAI-1 is observed by day 14 with a more important elevation occurring by day 21 in SOS patients.

Risk factors for SOS development are generally divided into patient-related factors (e.g., prior therapy, age, and preexisting liver disease) or transplantation-related factors (e.g., HLA-mismatched, conditioning regimen containing busulfan, cyclophosphamide, or TBI (total body irradiation)) [37, 42]. In our study, we found a statistically significant increase of SOS diagnosis in patients who received 3 or more previous lines of treatment before HSCT. We also observed a higher percentage of SOS patients who had received a previous autologous transplant. Both risk factors are derived from preexisting endothelial damage before HSCT. Association with other risk factors such as older age and busulfan-based conditioning are also observed in the SOS group [43].

Interestingly, two-thirds of the patients in the SOS group underwent a haploidentical HSCT, compared to only 33% in the non-SOS group. This result is not statistically significant and should be interpreted with caution given the small number of patients in the study. An increased incidence of SOS in HLA-haploidentical HSCT is expected given the high degree of HLA mismatch and the use of cyclophosphamide in the posttransplant setting [8]. Several retrospective studies have showed no increased incidence of SOS for haploidentical HSCT [5, 44]. However, no prospective study has yet specifically addressed the risk of SOS in this setting and a haploidentical donor is still considered a risk factor as any HLA-mismatched donor [16, 18, 22, 36].

Finally, we observed the evolution of biomarkers in SOS patients during treatment with defibrotide (Figure 3). PAI-1 kinetics resemble those of total bilirubin: we appreciate an important decrease after day 21, followed by a complete normalization of its levels after treatment with defibrotide by day 28 as already observed in a previous study [1]. On the other hand, HA and VCAM1 levels remained elevated even after SOS resolution, which could potentially limit its diagnostic role for a SOS relapse. Complete resolution of the inflammatory state at the hepatic sinusoids takes longer than restoration of the thrombotic-fibrinolytic balance. This would explain the slower decrease of HA and VCAM1 levels compared to PAI-1.

Furthermore, late-onset SOS can present without total bilirubin elevation [45] which can complicate its diagnosis even more [5]. The role of these biomarkers in the late-onset SOS remains to be determined but might also represent a useful tool in this setting to reach SOS diagnosis and eventually guide the duration of defibrotide treatment.

The small number of patients included in the study is an important limitation. Moreover, available samples on day 7 are limited, which potentially decreases the specificity and limits the interpretation of our results. None of our patients developed late-onset SOS, and our study does not address the possible use of these biomarkers in that setting [8], and this can potentially limit the interpretation of results. We report a SOS incidence of 14% for the whole study population (20% for allogeneic HSCT and 5.9% for autologous HSCT) which is considerably high compared to previously reported incidence [3, 36, 46]. Nevertheless, most studies report an incidence with a range that can go from 0% to 40% in allogeneic HSCT and 0% to 12% in autologous HSCT [2]. This variability from one center to another evidences the lack of accuracy of the existing SOS clinical criteria and stresses the need to discover new tools to improve SOS diagnosis. Our study includes both patients undergoing autologous and allogeneic HSCT, as previous studies focusing on SOS have previously reported [26, 29]. However, conditioning regimens used for autologous HSCT are generally less aggressive and its SOS incidence is lower than for allogeneic HSCT, which could also limit the interpretation of results.

## 5. Conclusion

In summary, our study shows that HA is a potential independent biomarker to help establish early SOS diagnosis even before other known signs and symptoms have been declared. The rise of VCAM1 and PAI-1 seems to take place in a later phase, together with total bilirubin rise.

Our findings support further investigation of the role of these 3 biomarkers in a multicenter setting. These studies should address the potential role of HA as a predictor of SOS and its potential incorporation to risk scores of SOS development [9, 30]. Future analyses should also question whether there is a significant increase in SOS incidence in the haploidentical HSCT setting.

## Figures and Tables

**Table 1:** Patient characteristics.

Patient's characteristics	SOS (*n* = 7)	Non-SOS (*n* = 40)	*p* value (Mann–Whitney/Fisher's exact)
Age (years, median (range))	59 (25-70)	51 (20-68)	0.198
Female sex, *n* (%)	3 (43%)	19 (48%)	0.999
Type of HSCT, *n* (%)			0.395
Autologous	1 (14%)	16 (40%)	
Allogeneic	6 (86%)	24 (60%)	
Type of donor, *n* (%)			
MUD	0	8 (20%)	0.155
MRD	2 (29%)	8 (20%)	0.999
Haploidentical	4 (57%)	8 (20%)	0.184
Conditioning regimen, *n* (%)			
Intensity			0.173
Myeloablative	3 (43%)	30 (75%)	
Nonmyeloablative	4 (57%)	10 (25%)	
Containing busulfan	5 (71%)	22 (55%)	0.682
Containing cyclophosphamide	4 (57%)	17 (43%)	0.684
Containing TBI	0	2 (5%)	0.999
Previous chemotherapy lines, median (range)	3 (2-9)	2 (1-5)	0.019
Previous autologous HSCT, *n* (%)	2 (29%)	3 (8%)	0.154
Hematologic disease, *n* (%)			0.986
Acute myeloid leukemia	2 (29%)	11 (27%)	
Acute lymphoid leukemia	0	5 (13%)	
Myelodysplastic syndrome	1 (14%)	3 (8%)	
Multiple myeloma	1 (14%)	4 (10%)	
Non-Hodgkin lymphoma	2 (29%)	9 (23%)	
Hodgkin lymphoma	1 (14%)	7 (18%)	
Aplastic anemia	0	1 (3%)	

MRD: matched related donor; MUD: matched unrelated donor.

**Table 2:** Number of patients predicted as having SOS (yes) or not (no) according to their HA level on day 7 (cutoff HA value > 172 ng/ml).

	SOS-yes	SOS-no
Predicts yes	7	7
Predicts no	0	9

Sensitivity = 100% (95% CI: 59.04-100.00). Specificity = 56.25% (95% CI: 29.88-80.25). SOS: sinusoidal obstruction syndrome; HA: hyaluronic acid.

**Table 3:** Number of patients with/without SOS according to the number of previous lines of treatment.

		SOS		Total
		Yes	No	
Previous treatments	<3 lines	2	29	31
	≥3 lines	5	11	16
Total		7	40	47

Fisher's exact test = 0.036. SOS: sinusoidal obstruction syndrome.

## Data Availability

The datasets used and analyzed during the current study are available from the corresponding author on reasonable request.

## Additional Points

*Novelty Statement.* (i) What is the *new* aspect of your work? Plasmatic levels of hyaluronic acid (HA) significantly increase before sinusoidal obstructive syndrome (SOS) clinical diagnosis. (ii) What is the *central* finding of your work? HA potentially represents an independent biomarker to guide SOS prophylaxis and treatment. (iii) What is (or could be) the *specific* clinical relevance of your work? SOS is a potentially fatal complication after hematopoietic stem cell transplant (HSCT), and therefore, new noninvasive methods to early diagnose SOS are needed.

## Supplementary Materials

Supplementary Materials Supplementary Figure 1: a ROC curve of HA predicting SOS at day 7 after allogeneic HSCT. Supplementary Table 1: the number of patients undergoing allogeneic HSCT predicted as having SOS or not according to their HA level on day 7. Supplementary Figure 2: the evolution of the mean of HA in patients with and without SOS in allogeneic and autologous HSCT patients.

## References

[1] Mohty M., Malard F., Abecassis M. (2015). Sinusoidal obstruction syndrome/veno-occlusive disease: current situation and perspectives--a position statement from the European Society for Blood and Marrow Transplantation (EBMT). *Bone Marrow Transplantation*.

[2] Coppell J. A., Richardson P. G., Soiffer R. (2010). Hepatic veno-occlusive disease following stem cell transplantation: incidence, clinical course, and outcome. *Biology of Blood Marrow Transplantation*.

[3] Dalle J. H., Giralt S. A. (2016). Hepatic veno-occlusive disease after hematopoietic stem cell transplantation: risk factors and stratification, prophylaxis, and treatment. *Biology of Blood and Marrow Transplantation*.

[4] Carreras E., Diaz-Ricart M. (2011). The role of the endothelium in the short-term complications of hematopoietic SCT. *Bone Marrow Transplantation*.

[5] Bazarbachi A. H., Al Hamed R., Labopin M. (2021). Underdiagnosed veno-occlusive disease/sinusoidal obstruction syndrome (VOD/SOS) as a major cause of multi-organ failure in acute leukemia transplant patients: an analysis from the EBMT acute leukemia working party. *Bone Marrow Transplantation*.

[6] Ragoonanan D., Khazal S. J., Wang J. (2021). Improved detection of sinusoidal obstructive syndrome using pediatric-AYA diagnostic criteria and severity grading. *Bone Marrow Transplantation*.

[7] Jones R. J., Lee K. S. K., Beschorner W. E. (1987). Venoocclusive disease of the liver following bone marrow transplantation. *Transplantation*.

[8] Mohty M., Malard F., Abecassis M. (2016). Revised diagnosis and severity criteria for sinusoidal obstruction syndrome/veno-occlusive disease in adult patients: a new classification from the European Society for Blood and Marrow Transplantation. *Bone Marrow Transplantation*.

[9] Strouse C., Zhang Y., Zhang M. J. (2018). Risk score for the development of veno-occlusive disease after allogeneic hematopoietic cell transplant. *Biology of Blood and Marrow Transplantation*.

[10] Ravaioli F., Colecchia A., Alemanni L. V. (2019). Role of imaging techniques in liver veno-occlusive disease diagnosis: recent advances and literature review. *Expert Review of Gastroenterology & Hepatology*.

[11] Chan S. S., Colecchia A., Duarte R. F., Bonifazi F., Ravaioli F., Bourhis J. H. (2020). Imaging in hepatic veno-occlusive disease/sinusoidal obstruction syndrome. *Biology of Blood and Marrow Transplantation*.

[12] Nishida M., Kahata K., Hayase E. (2018). Novel ultrasonographic scoring system of sinusoidal obstruction syndrome after hematopoietic stem cell transplantation. *Biology of Blood and Marrow Transplantation*.

[13] Iwai T., Nishida M., Sugita J. (2021). Reliability of an ultrasonographical scoring system for diagnosis of sinusoidal obstruction syndrome/veno-occlusive disease in patients with hematopoietic stem cell transplantation. *Journal of Medical Ultrasonics*.

[14] Nishida M., Sugita J., Takahashi S. (2021). Refined ultrasonographic criteria for sinusoidal obstruction syndrome after hematopoietic stem cell transplantation. *International Journal of Hematology*.

[15] Nishida M., Sugita J., Iwai T. (2022). Ultrasonographic scoring system of late-onset sinusoidal obstruction syndrome/veno-occlusive disease after hematopoietic stem cell transplantation. *Bone Marrow Transplantation*.

[16] Cairo M. S., Cooke K. R., Lazarus H. M., Chao N. (2020). Modified diagnostic criteria, grading classification and newly elucidated pathophysiology of hepatic SOS/VOD after haematopoietic cell transplantation. *British Journal of Haematology*.

[17] Dohan A., Guerrache Y., Boudiaf M., Gavini J.-P., Kaci R., Soyer P. (2014). Transjugular liver biopsy: indications, technique and results. *Diagnostic and Interventional Imaging*.

[18] Bonifazi F., Barbato F., Ravaioli F. (2020). Diagnosis and treatment of VOD/SOS after allogeneic hematopoietic stem cell transplantation. *Frontiers in Immunology*.

[19] Shulman H. M., Fisher L. B., Schoch H. G., Henne K. W., McDonald G. B. (1994). Venoocclusive disease of the liver after marrow transplantation: histological correlates of clinical signs and symptoms. *Hepatology*.

[20] Richardson P. G., Soiffer R. J., Antin J. H. (2010). Defibrotide for the treatment of severe hepatic veno-occlusive disease and multiorgan failure after stem cell transplantation: a multicenter, randomized, dose-finding trial. *Biology of Blood and Marrow Transplantation*.

[21] Corbacioglu S., Topaloglu O., Aggarwal S. (2022). A systematic review and meta-analysis of studies of defibrotide prophylaxis for veno-occlusive disease/sinusoidal obstruction syndrome. *Clinical Drug Investigation*.

[22] Mohty M., Malard F., Abecasis M. (2020). Prophylactic, preemptive, and curative treatment for sinusoidal obstruction syndrome/veno-occlusive disease in adult patients: a position statement from an international expert group. *Bone Marrow Transplant*.

[23] Chalandon Y., Mamez A., Giannotti F. (2022). Defibrotide Shows Efficacy in the Prevention of Sinusoidal Obstruction Syndrome After Allogeneic Hematopoietic Stem Cell Transplantation: A Retrospective Study. *Transplantation and Cellular Therapy*.

[24] Richardson P. G., Smith A. R., Kernan N. A. (2020). Pooled analysis of day 100 survival for defibrotide-treated patients with hepatic veno-occlusive disease/sinusoidal obstruction syndrome and ventilator or dialysis dependence following haematopoietic cell transplantation. *British Journal of Haematology*.

[25] Chalandon Y., Roosnek E., Mermillod B. (2004). Prevention of veno-occlusive disease with defibrotide after allogeneic stem cell transplantation. *Biology of Blood and Marrow Transplantation*.

[26] Cheuk D. K., Chiang A. K., Ha S. Y., Chan G. C., Cochrane Haematological Malignancies Group (2015). Interventions for prophylaxis of hepatic veno-occlusive disease in people undergoing haematopoietic stem cell transplantation. *Cochrane Database of Systematic Reviews*.

[27] Akil A., Zhang Q., Mumaw C. L. (2015). Biomarkers for diagnosis and prognosis of sinusoidal obstruction syndrome after hematopoietic cell transplantation. *Biology of Blood and Marrow Transplantation*.

[28] Zaid M. A., Wu J., Wu C. (2017). Plasma biomarkers of risk for death in a multicenter phase 3 trial with uniform transplant characteristics post–allogeneic HCT. *Blood*.

[29] Fried M. W., Duncan A., Soroka S. (2001). Serum hyaluronic acid in patients with veno-occlusive disease following bone marrow transplantation. *Bone Marrow Transplantation*.

[30] Jiang S., Penack O., Terzer T. (2021). Predicting sinusoidal obstruction syndrome after allogeneic stem cell transplantation with the EASIX biomarker panel. *Haematologica*.

[31] Luft T., Dreger P., Radujkovic A. (2021). Endothelial cell dysfunction: a key determinant for the outcome of allogeneic stem cell transplantation. *Bone Marrow Transplantation*.

[32] Hildebrandt G. C., Chao N. (2020). Endothelial cell function and endothelial-related disorders following haematopoietic cell transplantation. *British Journal of Haematology*.

[33] Paczesny S. (2018). Biomarkers for posttransplantation outcomes. *Blood*.

[34] Palomo M., Diaz-Ricart M., Carreras E. (2019). Endothelial dysfunction in hematopoietic cell transplantation. *Clinical Hematology International*.

[35] Van Den Broek M. A. J., Vreuls C. P. H., Winstanley A. (2013). Hyaluronic acid as a marker of hepatic sinusoidal obstruction syndrome secondary to oxaliplatin-based chemotherapy in patients with colorectal liver metastases. *Annals of Surgical Oncology*.

[36] Corbacioglu S., Jabbour E. J., Mohty M. (2019). Risk factors for development of and progression of hepatic veno-occlusive disease/sinusoidal obstruction syndrome. *Biology of Blood and Marrow Transplantation*.

[37] Bonifazi F., Sica S., Angeletti A. (2021). Veno-occlusive disease in HSCT patients: consensus-based recommendations for risk assessment, diagnosis, and management by the GITMO group. *Transplantation Lippincott Williams and Wilkins*.

[38] Lia G., Giaccone L., Leone S., Bruno B. (2021). Biomarkers for early complications of endothelial origin after allogeneic hematopoietic stem cell transplantation: do they have a potential clinical role?. *Frontiers in Immunology*.

[39] Zhang D., Yuan D., Shen J. (2015). Up-regulation of VCAM1 relates to neuronal apoptosis after intracerebral hemorrhage in adult rats. *Neurochemical Research*.

[40] Balakrishnan B., Illangeswaran R. S. S., Rajamani B. M. (2020). Prognostic plasma biomarkers of early complications and graft‐versus‐host disease in patients undergoing allogeneic hematopoietic stem cell transplantation. *eJHaem*.

[41] Westendorp R. G. J., Hottenga J. J., Slagboom P. E. (1999). Variation in plasminogen-activator-inhibitor-1 gene and risk of meningococcal septic shock. *Lancet*.

[42] Salat C., Holler E., Kolb H. J. (1997). Plasminogen activator inhibitor-1 confirms the diagnosis of hepatic veno-occlusive disease in patients with hyperbilirubinemia after bone marrow transplantation. *Blood American Society of Hematology*.

[43] Schechter T., Perez-Albuerne E., Lin T. F. (2020). Veno-occlusive disease after high-dose busulfan-melphalan in neuroblastoma. *Bone Marrow Transplantation*.

[44] Cai X., Wu J., Gui R. Y. (2019). Incidence, risk factors and outcomes of sinusoidal obstruction syndrome after haploidentical allogeneic stem cell transplantation. *Annals of Hematology*.

[45] Kaleelrahman M., Eaton J. D., Leeming D. (2003). Role of plasminogen activator inhibitor-1 (PAI-1) levels in the diagnosis of BMT-associated hepatic veno-occlusive disease and monitoring of subsequent therapy with defibrotide (DF). *Hematology*.

[46] Soyer N., Gunduz M., Tekgunduz E. (2020). Incidence and risk factors for hepatic sinusoidal obstruction syndrome after allogeneic hematopoietic stem cell transplantation: a retrospective multicenter study of Turkish hematology research and education group (ThREG). *Transfusion and Apheresis Science*.

