# Supplementary material for: Low Plasma Levels of Hyaluronic Acid Might Rule Out Sinusoidal Obstruction Syndrome after Hematopoietic Stem Cell Transplantation
Source: Dis Markers. 2023 Apr 17;2023:7589017. doi: 10.1155/2023/7589017 (PMC10125768; doi:10.1155/2023/7589017)
Supplement: Supplementary Materials — Supplementary Figure 1: a ROC curve of HA predicting SOS at day 7 after allogeneic HSCT. Supplementary Table 1: the number of patients undergoing allogeneic HSCT predicted as having SOS or not according to their HA level on day 7. Supplementary Figure 2: the evolution of the mean of HA in patients with and without SOS in allogeneic and autologous HSCT patients. [file 7589017.f1.docx]

**SUPPLEMENTARY MATERIAL**

Supplementary Figure 1. *ROC curve of HA predicting SOS at day 7 after allogeneic HSCT.* *AUC obtained from a ROC.*


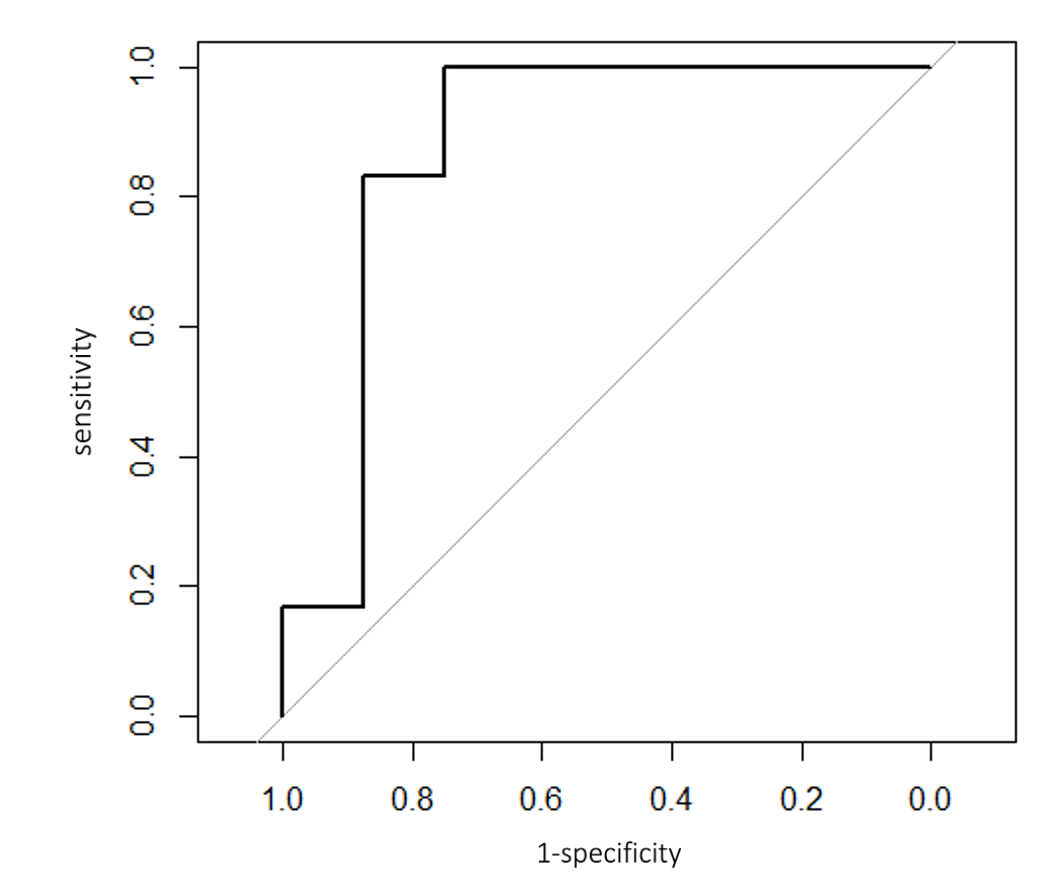


AUC = 0.875 (95%CI: 0.663 to 1)

*ROC: receiver operating characteristic; HA: hyaluronic acid; SOS: sinusoidal obstruction syndrome; HSCT: hematopoietic stem cell transplant; AUC: area under the curve.*

Supplementary Table 1. Number of patients undergoing allogeneic HSCT predicted as having SOS (yes) or not (no) according to their HA level on day 7 (cutoff HA value >172ng/ml).

|  | SOS-yes | SOS-no |
| --- | --- | --- |
| Predicts yes | 7 | 2 |
| Predicts no | 0 | 6 |
|  |  |  |

Sensitivity = 100% (95%CI: 54-100.00)

Specificity= 75% (95%CI: 35-97)

*SOS: sinusoidal obstruction syndrome; HA: hyaluronic acid; HSCT: hematopoietic stem cell transplant.*

Supplementary Figure 2. Evolution of the mean (standard deviations) of HA in patients with and without SOS in all patients, allogeneic HSCT patients and autologous HSCT patients.


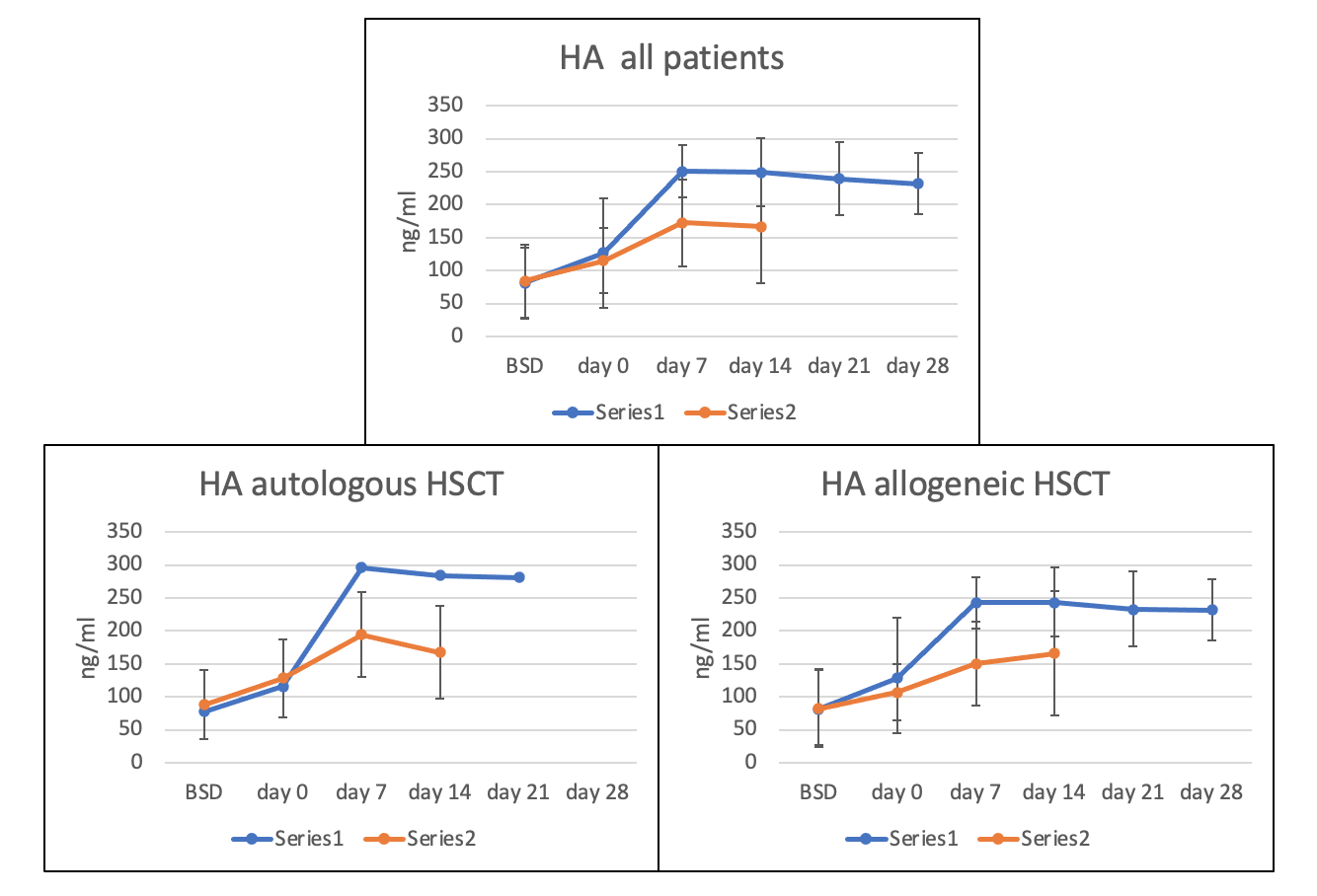


*SOS: sinusoidal obstruction syndrome; HSCT: hematopoietic stem cell transplant; HA: hyaluronic acid; BSD: basal sample day.*
